# Kinetic Analysis of High-Temperature Plastic Flow in 2.25Cr-1Mo-0.25V Steel

**DOI:** 10.3390/ma12244071

**Published:** 2019-12-06

**Authors:** Yongtao Zhang, Peng Luo, Longjiang Niu, Zhanpeng Lu, Haitao Yan, Xiaoli Hu

**Affiliations:** 1 School of Mechanical Engineering, Shanghai Dianji University, Shanghai 201306, China; zhangyt@sdju.edu.cn (Y.Z.); huxl@sdju.edu.cn (X.H.); 2 State Key Laboratory of Advanced Special Steel, Shanghai University, Shanghai 200444, China; zplu@t.shu.edu.cn; 3 Shanghai Collaborative Innovation Center for Heavy Casting/Forging Manufacturing Technology, School of Materials, Shanghai Dianji University, Shanghai 201306, China; luopeng@sdju.edu.cn; 4 Zhenshi Group Eastern Special Steel Co. Ltd., Jiaxing 314000, China; yanhaitao@zsess.com

**Keywords:** 2.25Cr-1Mo-0.25V steel, plastic flow, kinetics, hot tension mechanism

## Abstract

High-temperature plastic flow of heat-resistant 2.25Cr-1Mo-0.25V steel was investigated by hot tension (at 500–650 °C) on a Gleeble 3800 machine. The strain rate of hot tension was set as 0.001–1 s^−1^. The constitutive relation of the steel was modeled by the introduction of the parameters termed “true activation energy” and “threshold stress”. Then, the kinetics of high-temperature plastic flow was analyzed based on an Arrhenius equation modified by a “threshold stress”. The stress exponent of the modified equation was equal to 5. True activation energy was estimated to be 132 kJ·mol^−1^. According to the slip band model, the basic mechanism behind the hot deformation of the steel was considered to be dislocation climbing, which was governed by grain boundary diffusion. This model proved to be successful in its analysis of the experimental results of hot tension tests.

## 1. Introduction

As an iron-based alloy designed for high-temperature applications, the primary characteristic of 2.25Cr-1Mo-0.25V steel (in wt.%) is excellent strength at a high temperature. For example, a successful engineering application of this steel was reported about its application in the production of the components serving in chemical engineering, e.g., the vessels and pipes configured in hydro-cracking and hydro-desulfurization reactors [1]. For the materials used for these components, high temperature strength is one of the most important factors. It has been pointed out that excellent performances at high temperatures were derived from the strengthening effects caused by a large number of carbides precipitated by heat treatment. The strengthening effects depended on the classification and morphology of the carbides [2]. In addition, the understanding of the deformation and failure of steel at a high temperature is key in the design of the products for engineering applications. In this regard, it is of significance to understand plastic flow behavior and its underlying mechanism in order to design novel, heat-resistant Cr-Mo-V steel of long service life [3]. In particular, tensile testing is very useful to understand the constitutive correlation of materials. Thus, in this paper, hot deformation behavior of Cr-Mo-V steel was analyzed by tensile tests in considering the fact that most components of Cr-Mo-V steel would be stretched or roll-bended under a stress state resulting from a tension for technological service conditions. To date, a variety of works have been reported about the heat treatment, carbide evolution, and mechanical properties (e.g., creep life) of Cr-Mo-V steels [4,5,6,7]. Nevertheless, very few studies have been reported thus far about an instantaneous plastic flow of heat-resistant ferritic Cr-Mo-V steel at relatively low temperatures (500–650 °C) and there is a need to investigate the deformation mechanisms of plastic flow. Especially, the examination of the deformation behavior of Cr-Mo-V steels at the temperature ranging from 500 to 650 °C allow us to reveal the mechanisms governing the interaction between moving dislocations and dispersoids, and the mechanism controlling the rate of plastic deformation. Both mechanisms are sensitive to temperature. In addition, it is widely accepted that the rate of creep is highly sensitive to the stress in dispersion strengthened alloys, which suggests the existence of a threshold of stress—below which, the rate of creep become very slow [8]. Hence, an important issue worthy to be addressed is to consider a significant effect taken by a threshold stress on plastic flow behavior [9,10]. In this paper, high-temperature deformation was investigated by subjecting 2.25Cr-1Mo-0.25V steel (quenched and tempered) to hot tension tests on a Gleeble 3800 machine. The fundamental mechanism underlying the hot deformation of the steel was analyzed based on a kinetic model of plastic flow considering the activation energy and dislocation motion in the steel.

## 2. Materials and Methods

The chemical composition of 2.25Cr-1Mo-0.25V steel was determined by a SPECTRO optical emission spectrometry (OES, SPECTROLAB, Kleve, Germany) as 0.12C, 0.70Mn, 2.25Cr, 0.95Mo, 0.30V, <0.05Si, <0.012P, <0.003S and Fe balanced (in wt.%). An ingot of a mass of 150 kg was cast by pouring the melt (prepared by a vacuum-induction furnace) into a mold, and then hot-rolled to a sheet with ~20 mm in thickness. The operative norm of heat treatment for the sheet was described as follows: First, “austenization” was carried out at 940 °C for 0.5 h to achieve sufficient solution of carbon atoms. Second, the sheet was quenched (with water) to room temperature in order to maintain the constituent obtained at high temperature (a supersaturated solid solution of carbon atoms into iron matrix). Third, a tempering at 720 °C for 5 h was performed to achieve desired strength and ductility. The microstructure of 2.25Cr-1Mo-0.25V steel was inspected by scanning electron microscopy (SEM, JEOL S-4200 at 20 kV, Tokyo, Japan). A sample (in the form of thin film) was characterized by transmission electron microscopy (TEM, JEOL JEM-200CX at 160 kV, Tokyo, Japan) following the operative steps specified below: First, metallographic sample was mechanically ground, and then prepared by a twin-jet electro-polisher (Tenupol-5 Struer, at −20 °C and 30 V, Ballerup, Denmark) with a solution containing perchloric acid (5%) in ethanol. Further thinning was conducted by a Gatan precision ion-polishing system (PIPS model 691, Pleasanton, CA, USA) with Ar-beam energy of 4 keV at an incident angle of ±4°. For mechanical properties, quenched-tempered samples of a cylindrical shape (10 mm in diameter, and 120 mm in length) for tension test were machined from the hot-rolled plate. Hot tension tests were carried out at various strain rates (0.001, 0.01, 0.1 or 1 s^−1^) on a Gleeble 3800 machine (Poestenkill, NY, USA). The temperature at which tensile tests were conducted is 500, 550, 600 and 650 °C, respectively. The cylindrical samples (10 mm in diameter and 120 mm in height) of mechanical testing were heated at a heating rate of 5 °C s^−1^, and then held for 5 min to achieve homogeneous distribution of temperature. The specimens were hot tensioned until an ultimate failure was occurred. 

## 3. Results and Discussion

**Figure 1:**
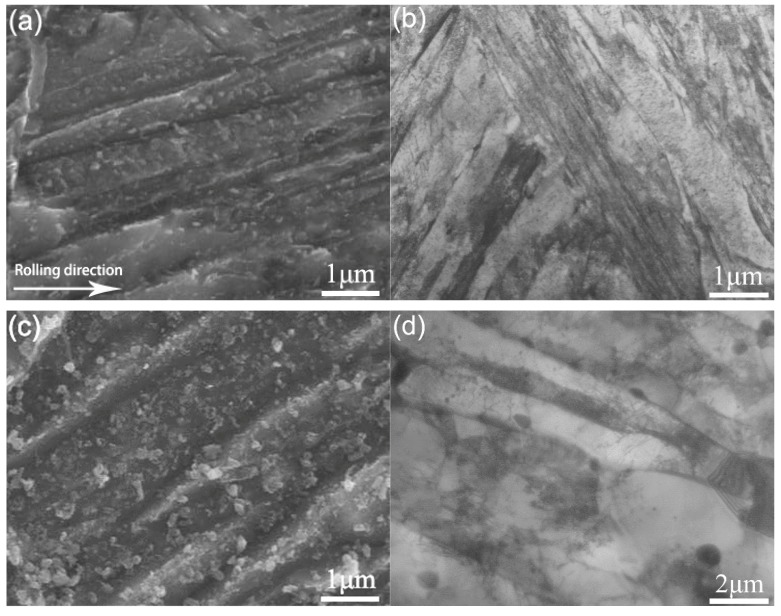
The microstructure of Cr-Mo-V steel by quenching: the image obtained from (**a**) SEM and (**b**) TEM, respectively; and the microstructure of the steel by tempering: the image from (**c**) SEM and (**d**) TEM, respectively.

The microstructure of the steel subjected to quenching is shown in Figure 1a,b. Lath-shaped martensites of high dislocation density were extensively existed. The width of the laths was 0.2–1 μm. An austenitic grain was divided into different domains. Each domain was taken as a group of laths having only one habit plane of given orientation. In other words, the laths in each domain were layered, sharing the same orientation of habit plane. The microstructure of the steel subjected to quenching and tempering (at 720 °C for 5 h) is shown in Figure 1c,d. The morphology of the tempered martensite was classified into two shapes, i.e., lath or equiaxed. Simultaneously, considerable amounts of precipitates were formed after tempering. There were two types of precipitates, i.e., coarse polygonal, and fine spherical, that were densely distributed at the boundaries between laths. The direction of rolling has been arrowed in Figure 1.

**Figure 2:**
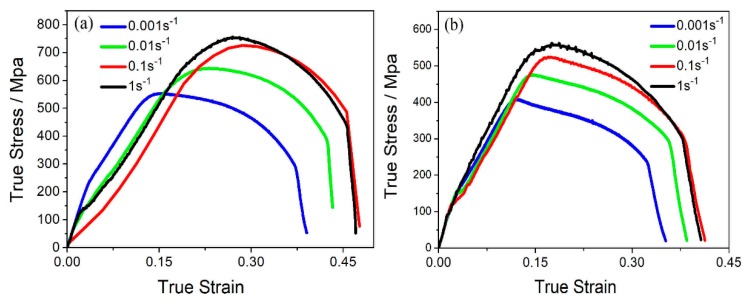
The stress–strain curves of 2.25Cr-1Mo-0.25V steel at various strain rates and two deformation temperatures: (**a**) 550 °C, and (**b**) 650 °C.

The temperature and strain rate of hot tension took remarkable effect on plastic flow. Figure 2 shows the curves true stress vs. true strain in 2.25Cr-1Mo-0.25V steel tensioned at temperature 550 or 650 °C and a strain rate 0.001–1 s^−1^. Obviously, the flow stress (and “peak stress”) at steady stage were increased with strain rate (This implied a strain-rate sensitivity of plastic flow behavior). According to the theory of dislocation kinetics, the relationship between flow stress (σ) and strain rate ($\dot{\varepsilon }$) was expressed by the following equations:

(1) $$\dot{\varepsilon }=K\rho bv,$$

(2) $$v=A\sigma^{m},$$

where, K is the ratio between the distances of dislocationsliding and climbing, ρ is the density of movable dislocations, b is the Burgers vector length, v is the average speed of dislocation motion, A is a constant, and m is the average value of Schmid factors [11]. According to these equations, a high strain rate resulted in large flow stress. This is because a large strain rate is synonymous with a quick deformation by which a pronounced strain-hardening effect was achieved by means of dislocation pile-up. Meanwhile, the degree of inconsistency between carbides and iron matrix was enhanced at large strain rate. Therefore, it was difficult to coordinate the deformations of carbides and iron matrix. As a result, the plastic flow (dislocation motion) of iron matrix was effectively hindered by carbides at quick deformation.

**Figure 3:**
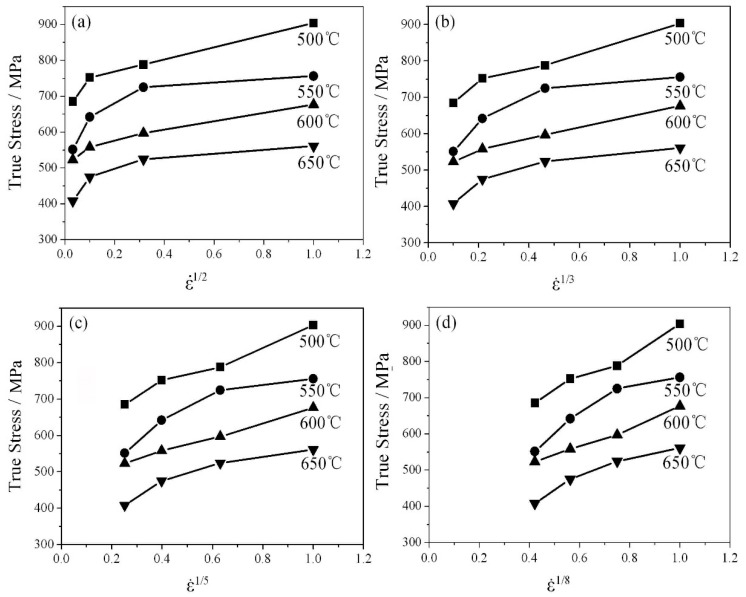
The curves true peak stress vs. ${\dot{\varepsilon }}^{1/n}$ at different deformation temperatures: (**a**) n = 2, (**b**) n = 3, (**c**) n = 5, and (**d**) n = 8.

**Figure 4:**
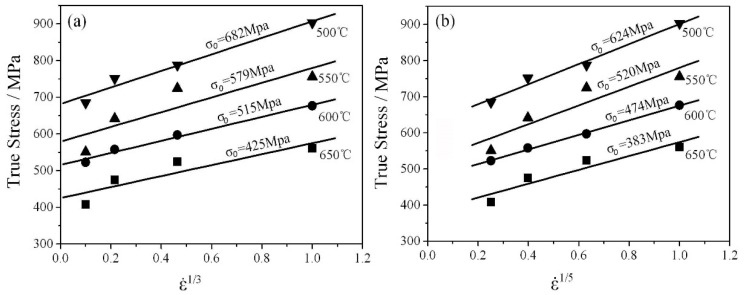
The regression true peak stress vs. ${\dot{\varepsilon }}^{1/n}$ at different temperatures: (**a**) *n* = 3, and (**b**) *n* = 5.

The kinetics of plastic flow was analyzed by considering the activation energy required to overcome the barrier for thermal activation at a high temperature. The Arrhenius equation is useful to describe high-temperature plastic flow because an instantaneous flow is analogous to high-temperature creep. The Arrhenius equation is given by [12]:

(3) $$\dot{\varepsilon }=A\sigma^{n}\exp\left(-\frac{Q_{a}}{RT}\right)$$

where, A is a constant, σ is the flow stress, n is the stress exponent, $Q_{a}$ is the apparent activation energy, T is deformation temperature, and R (=8.314 J·mol^−1^·K^−1^) is the molar gas constant. Although, as a classical phenomenological model, the Arrhenius equation is ideal for analyzing the plastic flow of single-phase alloy, it is not effective to unveil an obstacle effect of second phase on dislocation motion (deformation). That is to say, it is reluctant to propose the deformation mode of Cr-Mo-V steel by the classical Arrhenius equation because the phase constituent of the steel is very complex. There were considerable amounts of carbide precipitates formed in the steel during heat treating. To tackle this bottleneck, a threshold stress ($\sigma_{0}$) was introduced to modify the classical Arrhenius equation. In fact, $\sigma_{0}$ existed once an alloy was strengthened by dispersoids [13,14,15]. As a result, the Arrhenius equation was modified by substituting an effective stress ($\sigma -\sigma_{0}$) [13,14], i.e.,

(4) $$\dot{\varepsilon }=A^{\prime }{\left(\frac{\sigma -\sigma_{0}}{G}\right)}^{n}\exp\left(-\frac{Q_{t}}{RT}\right)$$

where $A^{\prime }$ is a constant, G is the shear modulus, and $Q_{t}$ is the “true” activation energy. By the aid of the modification, the Arrhenius equation is ideal for modeling plastic flow behavior at whatever low or high level of stresses [13,14]. According to this equation, the estimate of $\sigma_{0}$ is the prerequisite for determining $Q_{t}$. As shown in Figure 3, the line σ vs. ${\dot{\varepsilon }}^{\frac{1}{n}}$ (n = 2, 3, 5 and 8, corresponding to different deformation modes [15,16] was plotted by a standard linear extrapolation [17]. Consequently, $\sigma_{0}$ was extrapolated (at $\dot{\varepsilon }$ = 0) at various temperatures (500–650 °C) (as shown in Figure 4). In general practice, the parameter n = 2 means that a deformation is governed by grain boundary sliding. When n is 3, the deformation is derived from viscous dislocation glide. Simultaneously, the deformation may be caused by dislocation climbing once n is equal to 5. Moreover, if n = 8, the substructure of alloys maintains unvaried (This case is definitely unsuitable for Cr-Mo-V steel because its substructure was changed by hot tension, and thus the parameter n = 8 was inappropriate when the hot tension behavior of the steel was described). In this work, the value of n was estimated by a trial-and-error method. That is to say, only the best linearity was adopted by comparing all linear fittings σ vs. ${\dot{\varepsilon }}^{\frac{1}{n}}$. As shown in Figure 3, the linearity of the fitting was acceptable when n was only equal to 3 or 5. However, the case while n = 3 is irrational because with this value, $\sigma_{0}$ was larger than “peak stress” (see Figure 4a). Therefore, the parameter n = 5 was the only ideal for modeling the constitutive relation of Cr-Mo-V steel. This finding is especially consistent with the conclusion by Kassner that the regime corresponding to five-power-law creep is presented at higher stresses and lower temperatures, and this regime of creep is generally controlled by dislocation climb [18].

**Figure 5:**
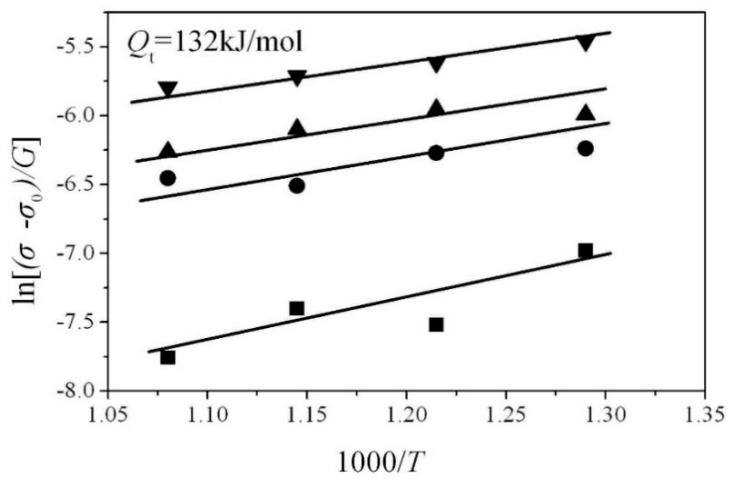
The plots $ln\left[\left(\sigma -\sigma_{0}\right)/G\right]$ vs. 1000/T.

According to a logarithmic transform for the modified Arrhenius equation, i.e.,

(5) $$ln\left(\frac{\sigma -\sigma_{0}}{G}\right)=\left(\frac{Q_{t}}{nR}\right)\left(\frac{1000}{T}\right)-\frac{1}{n}lnA^{\prime }+\frac{1}{n}ln\dot{\varepsilon }$$

the value of $Q_{t}$ (=132 kJ·mol^−1^) was estimated by plotting $ln\left(\sigma -\sigma_{0}\right)/G$ vs. 1000/T (as shown in Figure 5). This estimate is consistent with the activation energy (=140 kJ·mol^−1^) for grain boundary diffusion in α-Fe, as reported elsewhere [19].

**Figure 6:**
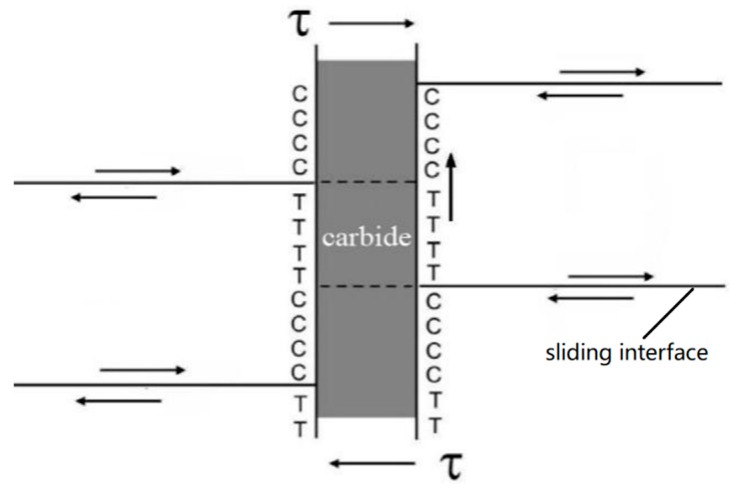
A modified slip band model exhibiting the carbides precipitated on grain/subgrain boundaries (The symbols C and T denote the region where compressive and tensile stresses are concentrated, respectively).

The slip band model was proposed by Spingarn and Nix [20] based on the theory of dislocation climbing at grain boundaries. Since grain boundary diffusion is much faster than lattice diffusion, climb rates are increased in the vicinity of a grain boundary. Hence, the slip band model is effective with an activation energy smaller than that of lattice diffusion, which provides a physical mechanism explaining the observation that activation energy is equal to the energy of grain boundary self-diffusion. According to this model, a tensile or compressive strain is produced in the vicinity of grain boundaries by a shear stress (externally applied). Both types of strains can be accommodated by grain boundaries in consideration of the fact that a vacancy flow is formed by the gradient of the concentration of vacancies between the regions tensioned and compressed (nearby grain boundaries). Following the theory of dislocation, the diffusional flow of vacancies is the cause for dislocation climb. In the slip band model, the diffusional flow of vacancies is occurring from the zone with a tensile stress to that with a compressive stress (and vice versa) [21]. This model provides a physical explanation of the phenomena as to how a self-diffusion at grain boundary was driven by a strong shear along slip bands, and how it was impeded by grain boundaries. In addition, the precipitation of carbides was observed in the vicinity of grain boundaries or subgrain boundaries (see Figure 1c,d). As a result, the pile-up of dislocations would be occurring near grain boundaries or subgrain boundaries. It is likely that slip bands generate near grain boundaries or subgrain boundaries [22,23]. Meanwhile, shear stresses are effectively transferred to grain boundaries owing to good rigidity of carbide precipitates [20]. Then, in the slip band model, tensile and compressive stresses can be transferred at different regions of grain boundaries. The process of transfer is driven by the difference in the concentrations of vacancies. This model is consistent with the result reported by Humphreys et al. [24] that the approaching dislocations climbed on the periphery of carbides. In this paper, the slip band model was modified in order to analyze the hot tension behavior of Cr-Mo-V steel (as shown in Figure 6). Namely, dislocations were pushed to grain or subgrain boundaries, and then hindered by the boundaries (and the particles precipitated on grain boundaries). Consequently, shear strains were produced at grain or subgrain boundaries. The process of hot deformation was governed by the coordination between the strain and dislocation climb at grain or subgrain boundaries (the diffusion of grain boundaries).

**Figure 7:**
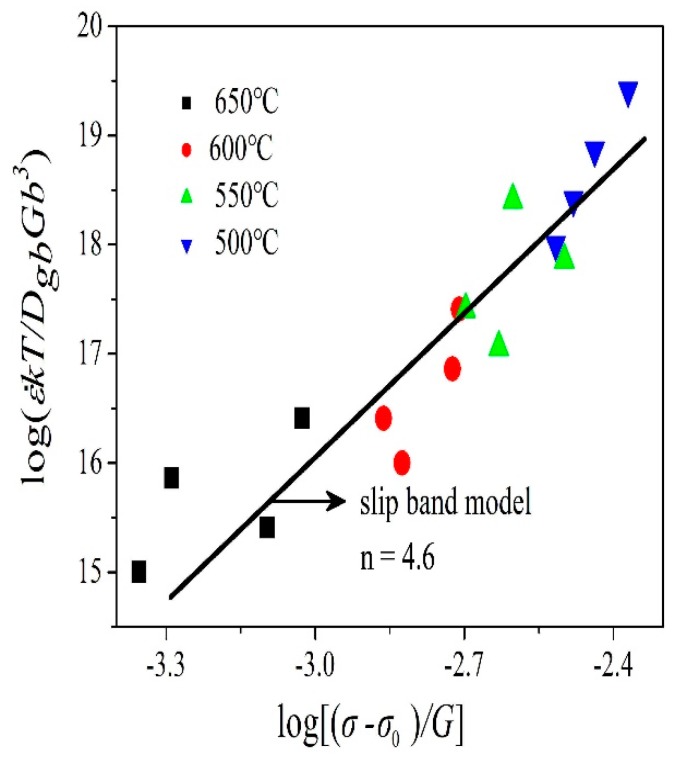
The comparison between the slip band model and experimental data: normalized strain rate vs. effective stress.

In addition, according to the slip band model, the estimate that n = 5 was verified by plotting $\left(\sigma -\sigma_{0}\right)/G$ vs. $\dot{\varepsilon }kT/D_{gb}Gb^{3}$ (where $D_{\mathrm{gb}}\left(=1.2\times 10^{12}\times e^{\frac{-140000}{8.314\times T}}\right)$ [19] is the coefficient of grain boundary diffusion, k (=1.380658 × 10^−23^) is the Boltzmann constant, b (=0.248 nm) is Burgers vector and the values of G are about 65.6–58.4 GPa [25]) on double logarithmic coordinates (as shown in Figure 7). The data of hot tension fitted well to the line with a slope estimated to be 4.6. Therefore, it is believable that the hot tension of the steel was governed by grain boundary diffusion. Furthermore, it ascertained again that the obtained experimental data of hot tension can be well satisfied with the slip band model proposed by Spingarn and Nix [20].

**Figure 8:**
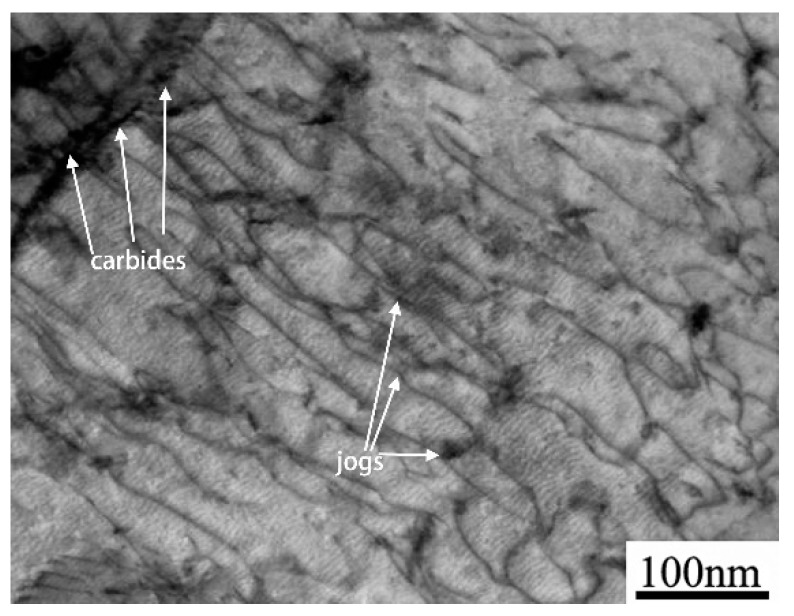
The interaction between dislocations and carbides in 2.25Cr-1Mo-0.25V steel.

**Figure 9:**
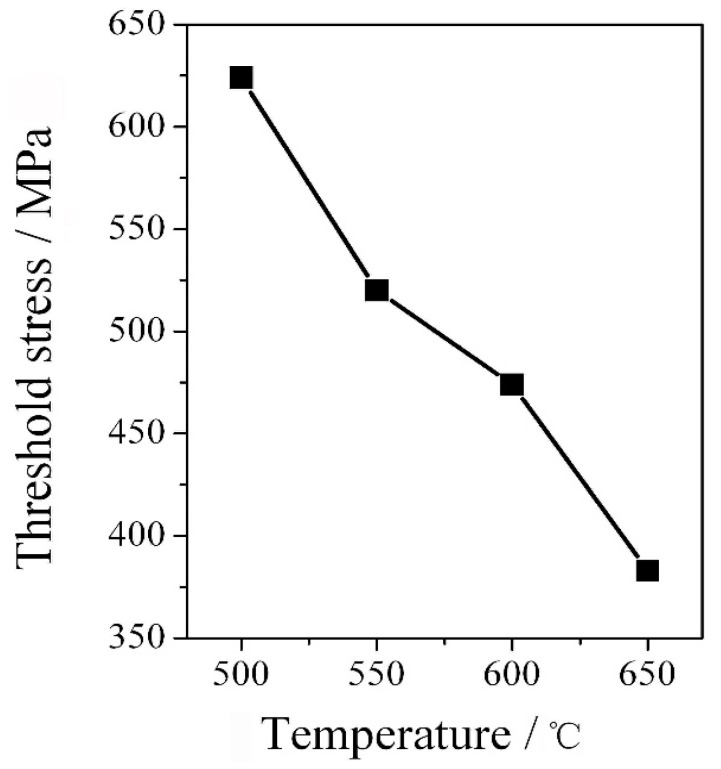
The plot threshold stress vs. deformation temperature in 2.25Cr-1Mo-0.25V steel.

As shown in Figure 8, a strong interaction of dislocations with carbides was verified by TEM. It is clear that the existence of some jogged screw dislocations (arrowed in Figure 8) is considered as an important evidence supporting a deformation mode corresponding to the five-power-law regime [26]. In addition, according to Ma and Tjong [17], a threshold stress was developed by the interaction between dislocation and second phase in aluminum matrix composite. Similarly, the threshold stress in Cr-Mo-V steel was defined by the obstacle effect caused by carbides. Also, a high level of stress-sensitivity of strain rate (i.e., a “threshold effect”) was observed in aluminum alloy strengthened by SiC dispersion [27]. Nevertheless, as indicated in Figure 9, the threshold stress was decreased with deformation temperature because the strength of the steel was largely impaired once deformation temperature was enhanced. On the other hand, the strengthening by the dispersion of fine carbides (i.e., the Orowan effect) was insensitive to temperature. This temperature-insensitivity obeys the Orowan–Ashby equation, as reported elsewhere [22,28].

The deformation of metals was preferably studied by in-situ SEM or in-situ TEM [29,30,31,32], which is impossible to be handled when dislocation motion is observed (for SEM) or the temperature of testing is much higher than 500 °C (for TEM). Instead, one can use classical Arrhenius equation established by trial-and-error method [15,17], which is phenomenological and empirical. 

## 4. Conclusions

High-temperature plastic flow of heat-resistant 2.25Cr-1Mo-0.25V steel was investigated by hot tension (at 500–650 °C) on a Gleeble 3800 machine. The strain rate of tensile tests varied from 0.001 to 1 s^−1^. The kinetics of plastic flow was analyzed based on an Arrhenius equation modified by a “threshold stress”. The stress exponent of the modified equation was equal to 5. The value of true activation energy was estimated to be 132 kJ·mol^−1^. The hot deformation of 2.25Cr-1Mo-0.25V steel was derived from dislocation climb governed by the diffusion of grain boundaries. A modified slip band model is successful in its analysis of the experimental results of hot tension.
